# Multiple Gene Deletion Mutants of Equine Herpesvirus 1 Exhibit Strong Protective Efficacy Against Wild Virus Challenge in a Murine Model

**DOI:** 10.3390/vaccines13010045

**Published:** 2025-01-08

**Authors:** Stephanie S. Pradhan, Vekataramireddy Balena, Bidhan Chandra Bera, Taruna Anand, Rhushikesh Khetmalis, Aashwina Madhwal, Supriya Kandasamy, Selvaraj Pavulraj, Manju Bernela, Priya Mor, Bhupendra Nath Tripathi, Nitin Virmani

**Affiliations:** 1 ICAR-National Research Centre on Equines, Sirsa Road, Hisar 125001, Haryana, India; steffi.pradhan05@gmail.com (S.S.P.); aashwina@outlook.com (A.M.); sruthi120995@gmail.com (S.K.); priyamor70@gmail.com (P.M.); 2 Division of Pathology, ICAR-Indian Veterinary Research Institute, Bareilly 243122, Uttar Pradesh, India; 3 Department of Pathobiological Sciences, School of Veterinary Medicine, Louisiana State University, Baton Rouge, LA 70803, USA; 4 Indian Council of Agricultural Research, Krishi Bhawan, New Delhi 110012, Delhi, India

**Keywords:** equine herpesvirus type 1 (EHV1), gene deletion mutants, modified live virus, pathogenicity, humoral immunity, cell-mediated immunity, mice model, vaccine efficacy

## Abstract

Background: Equine herpesvirus type 1 (EHV1) is a ubiquitous viral pathogen infecting the equine population worldwide. EHV1 infection causes respiratory illness, abortion, neonatal foal mortality, and myeloencephalopathy. The currently available modified live EHV1 vaccines have safety and efficacy limitations. The two mutant EHV1 viruses (vToH-DMV (∆IR6/gE) and vToH-QMV (∆IR6/UL43/gE/UL56)), generated by the deletion of genes responsible for virulence (gE and IR6) and immunosuppression (uL43 and uL56), have been previously characterized by our group and found to generate good immune responses. The present study aimed to determine the safety and protective efficacy of the above mutants against a virulent EHV1 challenge in a murine model. Methods: BALB/c mice were intranasally immunized with a live vToH-QMV or vToH-DMV vaccine. Intranasal booster immunization was given at 14 days post-vaccination (dpv). Both mutants induced an optimal level of EHV1-specific humoral and cell-mediated immune responses, as determined by virus neutralization assay, ELISA, and immunophenotyping. At 35 dpv, the mice were intranasally challenged with wild-type EHV1 (vRaj strain). Results: Amongst the two mutants, vToH-QMV induced a better immune response than the vToH-DMV vaccine. Furthermore, vToH-QMV provided good protection in mice against the virulent challenge. It specifically exhibited less severe clinical disease in terms of clinical signs, body weight reduction, and gross and histopathological lung lesions accompanied by early virus clearance. Conclusions: These studies are suggestive of vToH-QMV EHV1 being a potential vaccine candidate against EHV1 infection, which needs to be finally tested in the main host, i.e., horses.

## 1. Introduction

Equine herpesvirus type 1 (EHV1) is a ubiquitous viral pathogen of equines belonging to the genus *Varicellovirus* in the *Alphaherpesvirinae* subfamily. The viral infection in horses causes respiratory illness, late-term abortion, neonatal mortality, and neurological disorders [1]. EHV1 causes a more severe form of infection than EHV4 [2,3] and poses a major economic threat to the equine industry throughout the world, including India [4,5,6,7,8].

In EHV1-infected animals, the virus establishes a life-long latency in the trigeminal ganglionic neurons. Therefore, the eradication of EHV1 from a horse population is almost impossible in endemic regions. Nonetheless, effective vaccination, along with good managerial practices, can help in mitigating infections [3,9]. The current EHVl vaccines comprise the inactivated virus for the control of abortion in pregnant mares and respiratory disease and the modified live vaccine (MLV) for the prevention of respiratory disease [10,11]. Traditionally, inactivated vaccines have been in vogue, but they are known to produce only a humoral immune response, with limited clinical and virological protection and a requirement for repeated vaccination. These vaccines are not able to prevent cell-associated viraemia owing to a lack of cell-mediated immune responses [12]. The use of inactivated vaccines carries with it the risk that some infectious viral particles may survive the inactivation process and cause disease after administration to the animal. At present, two licensed MLV vaccines are available in the USA (Rhinomune^®^, by Boehringer Ingelheim, Duluth, GA, USA) and Europe (Prevaccinol^®^, by Rhway, NJ, USA) [13,14]. These MLVs have been developed from the naturally attenuated EHV1RacH strain, which was produced by passaging (256 nos) in porcine epithelial cells [15]. In general, attenuated live virus vaccines are preferred because they evoke a longer-lasting immune response. However, studies have also shown that available MLVs are unable to provide full protection against EHV1 infection, and immunity induced by natural infection is stronger than MLV. Despite widespread vaccination, outbreaks continue and remain a severe problem for the equine industry [13,15,16]. To date, no effective antiviral drugs or vaccines have been reported that can provide complete protection against EHV1 infections [1].

For effective control of the disease, there is a need for the development of a second-generation vaccine that stimulates humoral as well as cellular immune responses, similar to what is produced by natural infection. The new-generation genetic recombineering technologies allow the genomic manipulation of viruses’ successful attenuation, which has the potential to be used as a safer live vaccine candidate [12]. In this direction, various vaccine candidates using attenuated EHV1 strains, such as RacH, KyA, and NY03, have been developed and tested in animal models, including that of mice and horses, to assess their efficacy in inducing protection. [17,18,19,20,21]. However, only two modified live virus (MLV) vaccines based on the avirulent EHV1 strain RacH are commercially available, highlighting the challenges and progress in creating recombinant vaccines for the control of equine herpesvirus 1 infection in horses [22].

Previously, we constructed and tested EHV1 mutants developed by the deletion of specific genes (IR6, UL43, glycoprotein E, and UL56 genes) in combinations, i.e., vToH-DMV (∆IR6/gE) and vToH-QMV (∆IR6/UL43/gE/UL56), employing bacterial artificial chromosome (BAC)-based genetic recombineering. We have tested their level of attenuation, employing in vitro and in vivo studies for their suitability as modified live vaccine candidates [21]. In the present study, we aimed to determine the safety and protective efficacy of double- and quadruple-deletion mutant EHV1 vaccine candidates against a virulent EHV1 challenge in a murine model.

## 2. Materials and Methods

### 2.1. Virus

The highly pathogenic Rajasthan-98 strain (vRaj), isolated from an aborted fetus in Bikaner [5,23], and attenuated EHV1 mutants (vToH-DMV and vToH-QMV) [21], constructed and maintained in the equine pathology laboratory (ICAR-National Research Center on Equine, Hisar, India), were used for the in vivo challenge studies. The wild-type EHV1 parent Tohana strain (vToH), isolated from an aborted fetus, was used for the immunoassays [23]. All the virus stocks were propagated in Rabbit kidney 13 (RK-13) cells.

### 2.2. Ethical Statement

The necessary approvals for the mice experiments were obtained from the Institutional Animal Ethics Committee (IAEC) and Institutional Biosafety Committee (IBSC) via letter no NRCE/CPSCEA/2018-19, 7/2/2019 and NRCE/IBSC/13-08-2018, respectively. The mice were housed in the institute’s animal facility and were provided ad libitum feeding under standard conditions.

### 2.3. Experimental Design to Determine the Pathogenicity and Protective Efficacy of the vToH-DMV and vToH-QMV Vaccine Candidates in Mice

**Figure 1:**
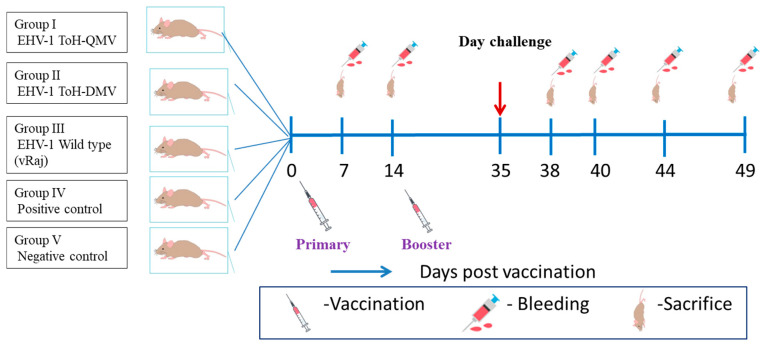
Schedule of vaccination and wild EHV1 challenge in BALB/c mice. Immunizations (primary and booster) were performed on the 7th and 14th days, respectively. Following vaccination, the challenge with wild EHV1 (vRaj) was conducted on the 35th day (arrow), and mice were sacrificed on the 7th, 14th, 35th, 38th, 40th, 44th, and 49th days post-vaccination.

Three- to four-week-old BALB/c mice (n = 124) were procured from the National Center for Laboratory Animal Sciences, National Institute of Nutrition, Hyderabad, India, and used for the in vivo study. The mice were randomly divided into five groups: 29 mice in each immunization group and 17 mice in both the positive and negative control groups. The mice were immunized intranasally with 1 × 10^7^ PFU/mouse (in 20 µL volume) of vToH-QMV (group I), vToH-DMV (group II), and wt vRaj (group III) under mild anesthesia (50 mg of Ketamine + 10 mg of Xylazine@100 mg per kg body weight, intraperitoneally). A booster dose was administered at 14 days post-vaccination (dpv) (Figure 1, Table S1). The control group animals (group IV, positive control (PC)) were mock immunized with 20 µL of sterile PBS. Three mice were sacrificed before inoculation on 0 dpv. Following immunization, three mice per interval were sacrificed from each group at 7, 14, and 35 dpv to study the immune response. At 35 dpv (0 day post-challenge; dpc), all mice were challenged intranasally with 1.5 × 10^7^ PFU/mouse of the wild-type (wt) vRaj (in 20 µL volume), except for the mice in the negative control (NC) group (group V), which were mock challenged with sterile PBS and sacrificed at 9 dpc. After the challenge, blood samples were collected from the retro-orbital plexus, and five mice per group were sacrificed at 3, 5, 9, and 14 dpc.

### 2.4. Sample Collection

For the humoral response, blood was collected in tubes without anticoagulant and allowed to clot at room temperature to obtain serum. The samples were centrifuged at 3000× *g* for 15 min, after which the serum was collected, aliquoted, and frozen at −80 °C until further use. To assess the cellular response by flow cytometry, whole blood was collected in a tube containing Ethylenediaminetetraacetic acid (EDTA).

### 2.5. Clinical Evaluation and Necropsy

Following immunization and the virus challenge, the mice in each group were monitored daily for clinical signs and body weight changes. The mice were sacrificed at predetermined intervals, as depicted in Figure 1, and a necropsy was performed. Any lesions observed were recorded and photographed. Gross lesion scoring was conducted with a maximum lesion score of 5 (+1 grade for congestion of lung parenchyma, 1–3 grades for red hepatization, and +1 grade for gray hepatization). Lungs and tissue samples (trachea and lung) were collected in 10% neutral-buffered formalin (NBF) for histopathological analysis and in sterile Hanks Balanced Salt Solution (HBSS) or viral transport medium for virological studies and then stored at −80 °C.

### 2.6. Evaluation of Immune Responses in Immunized Mice

#### 2.6.1. Estimation of Humoral Immune Response Using Serum Neutralization Assay

The EHV1-specific neutralizing antibody titers in the serum of the immunized mice were determined by a serum neutralization (SN) test at various intervals. Serum samples collected from three mice per group and were pooled and heat-inactivated at 56 °C for 30 min prior to testing. The processed serum samples were serially twofold diluted in the minimum essential medium (MEM) with 20% guinea pig complement in a 96-well plate. Subsequently, EHV1 antigen (@100 tissue culture infective dose 50; TCID_50_) was added to all the test wells and incubated at 37 °C for 3 h. Following incubation, RK-13 cells were added to each well, and the plates were incubated at 37 °C with 6% CO_2_. After four days of incubation, the cytopathic effect was observed, and the antibody titers were calculated as described previously [21,24]. The SNT assays were performed in triplicates for statistical analysis.

#### 2.6.2. Estimation of Humoral Immune Response by Indirect ELISA Test

The EHV1-specific antibodies in the serum of the mice from each group were determined using an indirect ELISA test. For the assay, purified EHV-1 (strain vToH) antigen [@ 1:1000 dilution (10^5.2^/mL)] was added in each well of an ELISA plate and incubated overnight at 4 °C, where we coated the plate. After incubation, the plates were washed with PBS-T (phosphate-buffered saline with 0.05% Tween 20) and blocked with 7.5% skimmed milk (#GRM 1254-500G, HiMedia, Thane West, India) followed by incubation at 37 °C for 1 h. The plates were then washed five times with PBS-T, and the test serum was added in twofold serial dilutions (starting from a 1:800 dilution) and incubated at 37 °C for 1 h. Subsequently, goat-anti-mouse HRPO conjugate (12349, Sigma Aldrich, MO, USA) at a 1:1000 dilution was added to each well, and the plate was incubated for one hour. After incubation, the substrate (3,3,5,5′ tetramethylbenzidine) (161018010A, Genei, Bangalore, India) was added to each well and incubated for 7 min. The substrate-enzyme reaction was stopped by the addition of 1 N sulfuric acid after an incubation period, and the absorbance at 450 nm was measured using an ELISA reader. The ELISA assays were performed in triplicates for statistical analysis.

#### 2.6.3. Determination of Isotyping of Antibody

The various mouse antibody subtypes, namely IgA, IgM, IgG1, IgG2a, IgG2b, and IgG3, were evaluated using an indirect ELISA with mouse monoclonal antibody isotyping reagents (ISO2, Sigma Aldrich, Bangalore, India). For the estimation of antibody subtypes, ELISA plates were coated with EHV-1 (strain vToH) antigen at a 1:500 dilution and incubated at 4 °C overnight. The coated plates were blocked with 7.5% skimmed milk for 1 h. Diluted serum samples in phosphate-buffered saline (1:800 dilution in PBS) were added to each well in duplicate and incubated at 37 °C for 1 h. Following incubation, plates were washed three times with PBS-T, and isotype-specific reagents (1:1000 diluted in PBS) were added into each well, followed by another incubation at 37 °C for 1 h. Subsequently, peroxidase-labeled rabbit anti-goat IgG (A-9169, Sigma Aldrich, Bangalore, India) diluted 1:15,000 was added to each well, followed by treatment with substrate-3,3′,5,5′ tetramethylbenzidine (161018010A, Genei, Bangalore, India). The color change resulting from the enzyme–substrate reaction was measured at 450 nm using an ELISA reader [21].

#### 2.6.4. Estimation of CD4+ and CD8+ Lymphocyte Populations by Flow Cytometry

**Figure 2:**
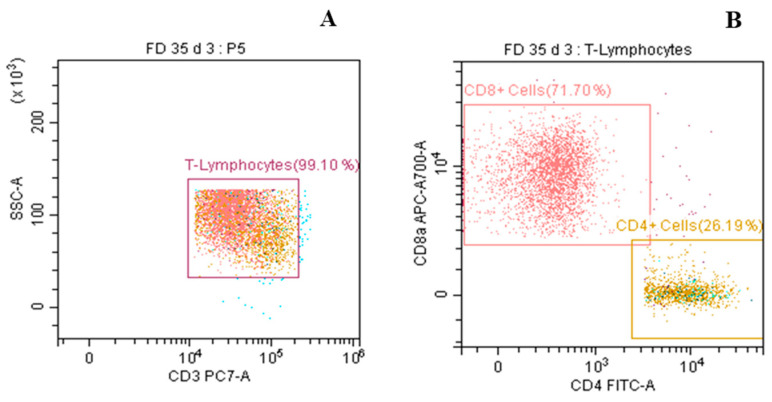
Immunophenotyping of CD4+ and CD8+ lymphocyte populations of experimental mice by flow cytometry. (**A**) Total lymphocytes were gated as CD3+ on a cytogram in the form of a dot plot. (**B**) CD4+ and CD8+ cells were further identified from CD3+ cells for immune response evaluation.

For immunophenotyping, peripheral blood mononuclear cells (PBMCs) were isolated from the whole blood using the RBC lysis method. The purified PBMCs were then immune-stained with CD3+, CD4+, and CD8+ specific reagents (Invitrogen, Waltham, MA, USA) following standard protocols. The stained samples were analyzed using a flow cytometer (Cytoflex, Beckman Coulter, CA, USA) to estimate the target cell population. In the cytoflex system, a total of 100,000 events were recorded and processed. Plots and gates were generated using appropriate gating controls. At the start of the experiment, compensation was performed using UltraComp compensation beads (01-2222-41, Invitrogen, Waltham, MA, USA), and the same settings were applied during data analysis. Cellular debris was excluded by gating, and a gated set was used to determine the lymphocyte population based on forward scatter (cell size) and side scatter (complexity or granularity). Subsequently, CD3+ T-cells were gated from the total lymphocytes on a cytogram displayed as a dot plot, followed by the identification and estimation of CD4+ and CD8+ cells within the CD3+ T-cells population (Figure 2) [21].

### 2.7. Histopathology

The nasal turbinate, trachea, and lung tissues were fixed in 10% NBF for histopathological examination. The fixed tissues were processed using a conventional method, embedded in paraffin, and sectioned at a thickness of 3–4 μm. These sections were then stained with Haematoxylin and Eosin (H&E) [25] for general histomorphology. Microphotography was performed using the Nikon Eclipse 80*i* microscope (Nikon Instruments Inc., Melville, NY, USA). The grading of histopathological lesions was carried out according to the criteria established by Pavulraj et al. [26], with minor modifications.

### 2.8. Detection of EHV1 Antigen by Indirect Immunoperoxidase Test (IIPT)

To confirm the presence of EHV1 antigen in the lung tissues of mice, the indirect immunoperoxidase test (IIPT) was performed on tissue sections using hyperimmune serum against whole EHV1 virus, which was raised in rabbits (at NRCE, Hisar) following the methodology described previously [21,27]. Briefly, the tissue sections were sequentially processed in 3% H_2_O_2_ solution in methanol, followed by treatment with trypsin (1 mg/mL) and calcium chloride solution (1 mg/mL) for 30 min at 37 °C. The tissue sections were then incubated with primary rabbit EHV1 hyperimmune serum (1:200 dilution) at 37 °C for 1 h, followed by incubation with goat anti-rabbit HRPO conjugate (A-9169, Sigma Aldrich, Bangalore, India; 1:200 dilution). The immunoreaction was developed using substrate-3, 3′-diaminobenzidine tetrahydrochloride (D 5905, Sigma Aldrich, Bangalore, India), and H_2_O_2_ as the substrate. Finally, the tissue sections were counterstained with 10% Harris Haematoxylin stain, and the stained EHV1 antigen was examined under a microscope.

### 2.9. EHV1 Viral Load Quantification by Quantitative PCR (qPCR)

Total viral DNA was isolated from nasal washings and lung tissue homogenates using the QIAamp^®^ DNA Blood Mini Kit (Qiagen, Hilden, Germany) and the QIAamp^®^ DNA Mini Kit (Qiagen, Hilden, Germany), respectively, following the manufacturer’s instruction. The quality and quantity of the purified DNA were assessed using a QIAxpert spectrophotometer (Qiagen, Hilden, Germany) and stored at −20 °C for further analysis. The purified viral DNA was then subjected to TaqMan probe-based qPCR targeting the ORF30 gene of EHV1, and the copy numbers were quantified using standard quantification methods. Viral DNA detection was performed using specific primers (forward primer: GTGGACGGTACCCCGGAC; reverse primer: GTGGGGATTCGCGCCCTCACC) and probes (FAM-ATCCGTCAACTACTCG-BHQ1). A standard curve was generated by qPCR using tenfold serial dilutions (10^2^ to 10^5^ copies) of plasmid DNA containing the cloned ORF30 region of EHV1 to calculate the copy number of the EHV1 virus in samples [28]. EHV1 standards and template DNA were run in duplicate on a real-time PCR machine (StepOne, Applied Biosystem, Waltham, MA, USA). The amplification program included an initial denaturation step at 95 °C for 5 min, followed by 40 cycles of denaturation at 95 °C for 15 s, annealing at 60 °C for 30 s, and extension at 60 °C for 1 min.

### 2.10. Statistical Analysis

All data were analyzed using GraphPad PRISM^®^ software version 8.0.2. A two-way analysis of variance (ANOVA) and Dunett’s test were performed to compare the mean values of the replicates for each parameter at different time points. A *p*-value of ˂0.05 was considered statistically significant.

## 3. Results

### 3.1. Mice Immunized with the Mutant EHV1 Exhibited Higher EHV1-Specific Serum Neutralization Antibody Titers Compared to the Wild-Type Group

**Figure 3:**
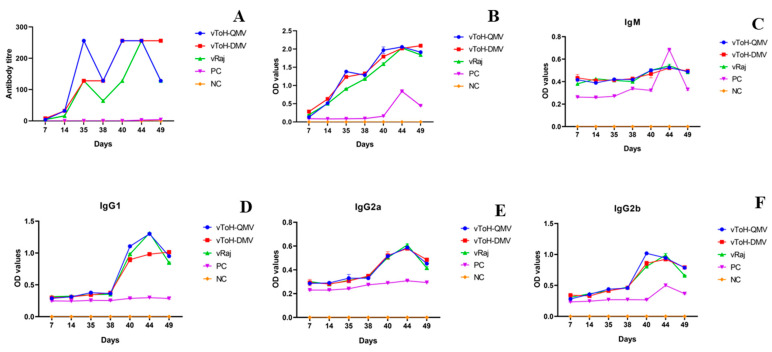
Humoral immune response following immunization and challenge in mice: (**A**) Serum neutralizing antibody titers; (**B**) estimated optical density values of ELISA (antibody titer); (**C**) serum IgM antibody subclass response; (**D**) serum IgG1 antibody subclass (IgG) response; (**E**) serum IgG2a antibody subclass (IgG) response; and (**F**) serum IgG2b antibody subclass (IgG) response.

Serum samples collected at various intervals were used to determine the EHV1-specific neutralizing antibody titers. Antibody responses were detectable starting from 7 dpv, with the titers ranging from 1:4 to 8, which increased to 1:16–32 by 14 dpv in all immunized groups (Figure 3A, Table S2). On the challenge day (35 dpv), all immunized mice from the three groups exhibited a significant increase in the antibody titers: 1:256 for vToH-QMV, 1:128 for vToH-DMV, and 1:128 for the wt group. Upon challenge, at 3 and 5 dpc, the antibody titers were 64 and 128 for the wt group, whereas they were 128 and 256 for both mutant groups, respectively. All immunized groups showed a titer of 1:256 at 9 dpc. In contrast, mice in the unvaccinated (day zero) and NC groups were serologically negative, while the PC mice showed a titer of 1:2 and 1:4 at 9 and 14 dpc, respectively (Table S2).

The results of the serum neutralization assay were further validated by indirect ELISA, which confirmed seropositivity against EHV1 by 14 dpv. The antibody levels progressively increased with a subsequent booster dose, and all three immunized groups showed a highly significant (*p* < 0.0001) elevation in OD values compared to the unvaccinated mice at 35 dpv (OD values: 1.38 ± 0.00, 1.23 ± 0.01, and 0.91 ± 0.01 for vToH-QMV, vToH-DMV, and wt, respectively) (Figure 3B, Table S3). Upon the challenge with the wt virus, the antibody responses were significantly increased. At 3 dpc, the OD values were 1.33 ± 0.00, 1.29 ± 0.01, and 1.18 ± 0.01 for vToH-DMV, vToH-QMV, and wt, respectively. The PC group did not show a significant OD value until 3 dpc, but it gradually increased from 0.16 ± 0.00 at 5 dpc to 0.84 ± 0.01 at 9 dpc (Table S3).

The humoral immune response was further characterized by assessing the immunoglobulin subclasses. The most prevalent antibody isotype observed after primary immunization was IgM, followed by IgG2b, while IgA could not be detected (Figure 3C–F). After booster immunization, antibody responses, particularly those of the IgG subclasses, showed a significant increase. IgG2b was found to be the most predominant. A significant surge in the OD values of all antibody subclasses was observed in the immunized mice following the challenge, starting from 5 dpc onwards. IgG1 was found to be the most dominant class (OD levels: 1.30 ± 0.01, 0.98 ± 0.01, and 1.31 ± 0.01 for vToH-QMV, vToH-DMV, and wt, respectively, at 9 dpc), followed by IgG2b (OD values: 0.94 ± 0.00, 0.92 ± 0.01, and 0.99 ± 0.03 for vToH-QMV, vToH-DMV, and wt, respectively, on 9 dpc), and IgG2a (Figure 3C–F). Notably, the highest IgG response was observed in the vToH-QMV group among the immunized groups. In contrast, the positive control group showed no EHV1-specific antibodies in the serum before the challenge. However, following the challenge, a significant increase in IgM (0.68 ± 0.00) and IgG2b (0.50 ± 0.00) was observed in the PC group only at 9 dpc.

### 3.2. Cell-Mediated Immune Response Against a Virulent EHV1 Challenge

**Figure 4:**
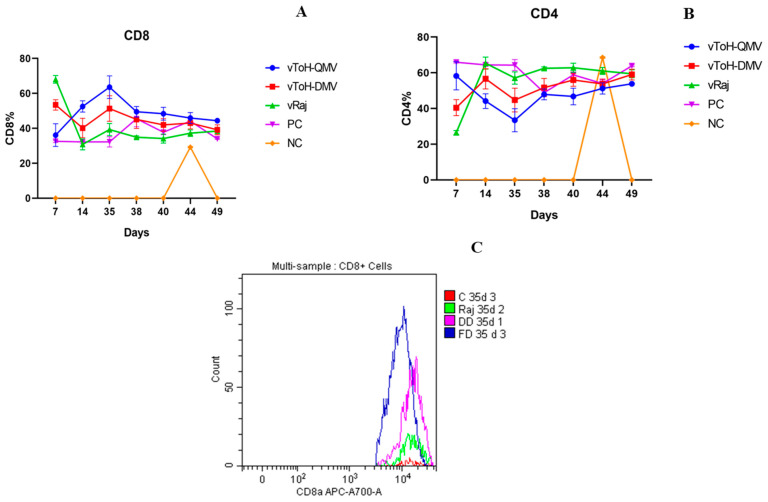
Cell-mediated immune response following immunization and challenge. (**A**) CD8 response of mice infected with different viruses at various intervals are depicted; (**B**) CD4 response of mice infected with different viruses at various intervals are depicted. NC animals that were sacrificed only on 9 dpc showed normal CD4:CD8 ratio (2:1); (**C**) Histogram overlay showing CD8 response of various groups at 35 dpv. The marked groups are as follows: red—PC, green—vRaj, pink—vToH-DMV, and blue—vToH-QMV.

On 0 dpv, mice exhibited a normal CD4/CD8 ratio (>2:1), with the CD4 cells dominating the population (Table S4). Similarly, the non-immunized control showed no rise in the CD8 population until the challenge. In contrast, by 7 dpv, mice immunized with wt showed a predominant CD8 population (68.04 ± 2.23%), which was significantly higher compared to the other groups (Figure 4A,B, Table S5). However, by 14 dpv, a significant decline was observed (30.83 ± 3.16%), followed by an increase at 35 dpv (39.27 ± 3.49%). Likewise, the mice from the vToH-DMV vaccinated group demonstrated a mild increase (53.41 ± 2.98%) in the CD8 response at 7 dpv followed by a decline at 14 dpv (40.20 ± 5.59%), which was not statistically significant. At the time of the challenge (35 dpv), the mean CD8 percentage increased again to 51.31 ± 7.30%. On the other hand, the vToH-QMV revealed a mild increase in the cellular immune response at 7 dpv (36.12 ± 6.52%), which was significantly lower compared to the response in the wt (*p* < 0.0001) and vToH-DMV groups (*p* < 0.01). Conversely, the response increased further at 14 dpv, with CD4 and CD8 percentages of 44.14 ± 4.08% and 52.52 ± 3.22%, respectively (Tables S4 and S5). The maximum response was detected at the time of challenge (35 dpv), where the average CD8 percentage reached 63.56 ± 6.45%, which was significantly higher than the response at 7 dpv (*p* < 0.0001) (Figure 4C). A significant statistical difference was also observed when compared to the wt and positive control groups (*p* < 0.0001).

Upon challenge, a gradual decline in the CD8 percentage, accompanied by an increase in the CD4 percentage, was observed in all groups. Notably, the highest CD8 population was depicted in vToH-QMV from days 3 through 14 post-challenge compared to the other groups. At 3 and 5 dpc, the CD8 values were statistically significant in the vToH-QMV group when compared to the wt group. By 14 dpc, CD4 cells become the dominant population.

### 3.3. Assessment of Clinical Signs and Body Weights in Mice upon Immunization and Challenge

Following primary immunization, clinical signs were more pronounced in the mice of the wild-type group, with their symptoms progressively declining from 9 dpv onwards. The mice exhibited anorexia, dehydration, moderate to severe piloerection, a ruffled coat appearance, huddling in the corners, mild to moderate dyspnoea, and unresponsiveness to any external stimuli between days 2 and 7 post-vaccination. In contrast, mice from the vToH-QMV and vToH-DMV groups showed mild to moderate clinical signs, after which they regained their vigor by 3 and 6 dpv, respectively. Following booster immunization, mice from all the groups remained normal. After the challenge, mice from the positive control group showed the most severe clinical signs. The severity of these clinical signs decreased from 6 dpc, and by 8 dpc, the mice appeared active and normal. No mice died during the experiment. Among the immunized groups, mice from the vToH-QMV group remained normal and healthy throughout the experiment. However, the mice from the vToH-DMV and vRaj displayed mild dyspnoea and piloerection but returned to their normal activities by 5 dpc.

**Figure 5:**
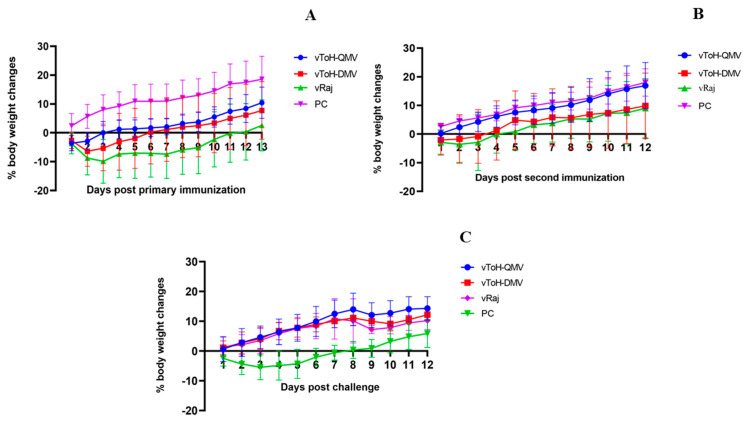
Mean body weight changes for each group are shown as the percentage. Body weight loss or gain in mice was monitored and recorded: (**A**) post-primary immunization, (**B**) secondary immunization, and (**C**) challenge. The mean body weight ± SD in each experimental group was determined at each time point.

The reduction in the mice’s body weights corroborated with the clinical signs. After primary immunization, significant weight loss was observed in the wt group. The weight loss peaked at 3 dpv (−9.86 ± 7.59), after which the mice began to regain weight by 12 dpv. In contrast, a minimal weight reduction was observed in the vToH-QMV and vToH-DMV groups on 1 dpv (−3.59 ± 2.63) and 2 dpv (−6.39 ± 5.23), respectively (Figure 5A,B, Tables S6 and S7). After the challenge, no weight reduction was observed in any of the three immunized groups (vToH-QMV, vToH-DMV, or wt) until the end of the study. In contrast, mice from the positive control group showed consistent weight loss, which peaked at 3 dpc (−5.46 ± 4.16) and continued until 7 dpc, after which their pre-challenge body weights were restored (Figure 5C, Table S8). The weight loss in the positive control group was highly significant when compared to the vToH-QMV, vToH-DMV, and wt groups (*p* < 0.0001).

### 3.4. Pathological Changes Exhibited by EHV1 Mutants Vis-à-Vis Wt Post-Challenge

**Figure 6:**
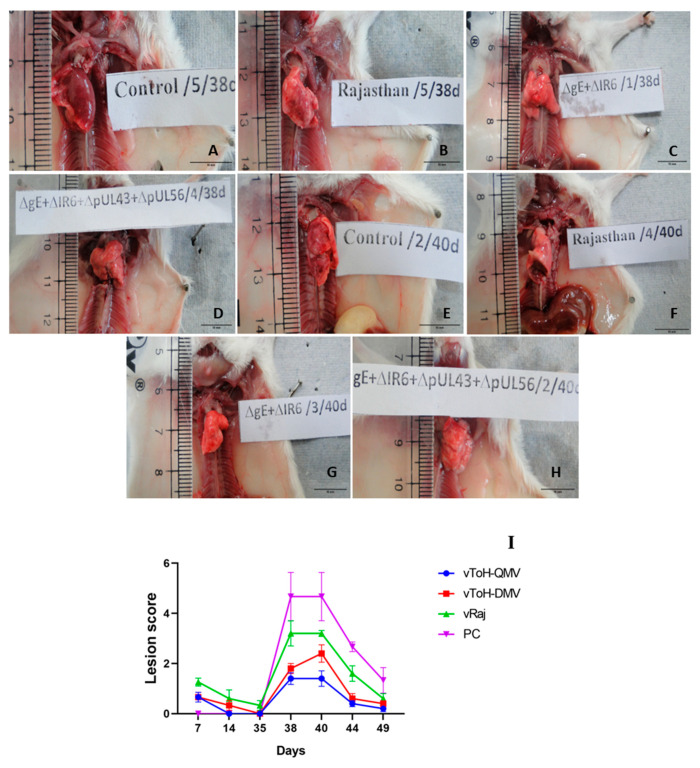
Pathogenicity of various strains at 3 and 5 dpc. (**A**) PC showing diffuse severe dark red areas (red hepatization) involving both the lungs; (**B**) vRaj showing focal widespread moderate red gray areas on the dorsal surface of the left lung; (**C**) vToH-DMV and (**D**) vToH-QMV showing mild to moderate areas of congestion; (**E**) PC showing moderate to severe red gray areas (gray hepatization) on the dorsal surface of the right lung; (**F**) vRaj showing gray area on the dorsal surface of left lung; (**G**) vToH-DMV and (**H**) vToH-QMV showing mild to moderate congestion. (**I**) Overall gross score of lung lesions at various intervals in different groups of mice.

Gross lesions were most noticeable in the lungs. At 3 dpc, the lungs of mice from the PC group showed moderate to severe multifocal to coalescing well-demarcated dark red areas of red hepatization, which gradually progressed to gray hepatization from 5 dpc onwards (Figure 6A,E, Table S9). However, signs of resolution were evident at 14 dpc. In contrast, the immunized groups exhibited similar lesions but with reduced severity in both intensity and magnitude. Among the immunized mice, the wt group mice exhibited lesions on the higher side until 9 dpc (Figure 6B,F) as compared to the mutant groups, which showed apparently healthy lungs (Figure 6C,D,G,H). Nevertheless, mice from the negative control group showed pink, healthy lungs. The overall gross score was highest for the PC group and lowest for the vToH-QMV group (Figure 6I, Table S9).

**Figure 7:**
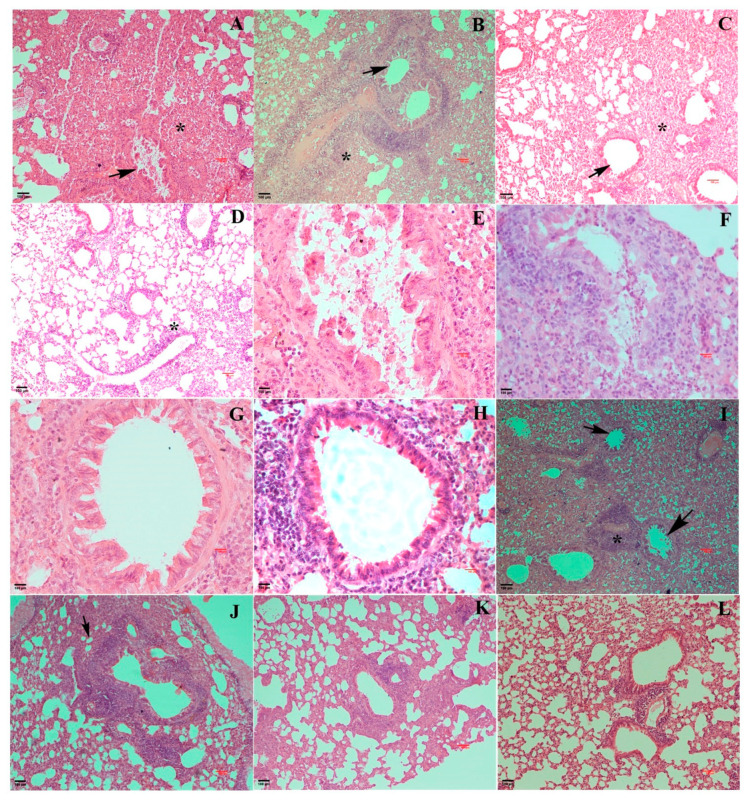

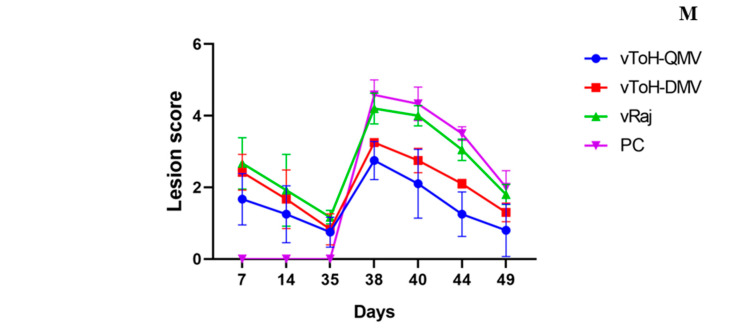
Microphotographs of lungs of mice. (**A**) Microphotograph of lung from PC showing severe multifocal to diffuse consolidation (*) and necrotic bronchiolitis (arrow) at 3 dpc (H&E 100X); (**B**) microphotograph of lung from vRaj showing moderate to severe consolidation (*) and bronchiolar necrosis and cuffing (arrow) at 3 dpc (H&E 100X); (**C**) microphotograph of lung from vToH-DMV showing moderate consolidation (*) and bronchiolar degeneration (arrow) at 3 dpc (H&E 100X); (**D**) microphotograph of lung from vToH-QMV showing multifocal mild to moderate consolidation (*) and mild bronchiolar degeneration at 3 dpc (H&E 100X); (**E**) microphotograph of lung from PC showing marked bronchiolar degeneration, necrosis, and desquamation with necrotic debris in the lumen at 3 dpc (*) (H&E 400X); (**F**) microphotograph of lung from vRaj showing severe bronchiolar degeneration and necrosis with desquamated debris admixed with neutrophils and fibrin in the lumen (*) at 3 dpc (H&E 400X); (**G**) microphotograph of lungs from vToH-DMV and (**H**) vToH-QMV showing mild to moderate bronchiolar necrosis at 3 dpc (H&E 400X); (**I**) microphotograph of lung from PC showing severe necrotizing alveolitis and bronchiolitis (arrow) accompanied with perivascular cuffing at 5 dpc (*) (H&E 100X); (**J**) microphotograph of lung from vRaj showing moderate multifocal interstitial thickening around the bronchioles (arrow) at 5 dpc (H&E 100X); (**K**) microphotograph of lung from vToH-DMV showing mild to moderate interstitial thickening, especially in the vicinity of the bronchioles and vessels at 5 dpc (H&E 100X); (**L**) microphotograph of lung from vToH-QMV showing mild interstitial thickening at 5 dpc (H&E 100X); (**M**) overall histopathological scores of lung lesions at various intervals in different groups of mice.

The histopathological changes were consistent with the gross findings. The positive control mice showed the most pronounced pathological changes in the lungs at 3 and 5 dpc (Figure 7A,I). Moderate to severe multifocal thickening of the interalveolar septa was evident primarily due to the severe engorgement of the pulmonary vasculature, proliferation of type 2 pneumocytes, and moderate to severe infiltration by a mixed population of neutrophils and macrophages. Among these, neutrophil infiltration was more prominent, gradually being surpassed by lymphocytes at the later stages of infection. Additionally, necrotizing alveolitis and bronchiolitis were observed, characterized by the presence of necrotic debris in the alveolar and bronchiolar lumen (Figure 7E). The margination of inflammatory cells along with the endothelial wall was notable, accompanied by perivascular peribronchiolar inflammatory cell cuffs.

**Figure 8:**
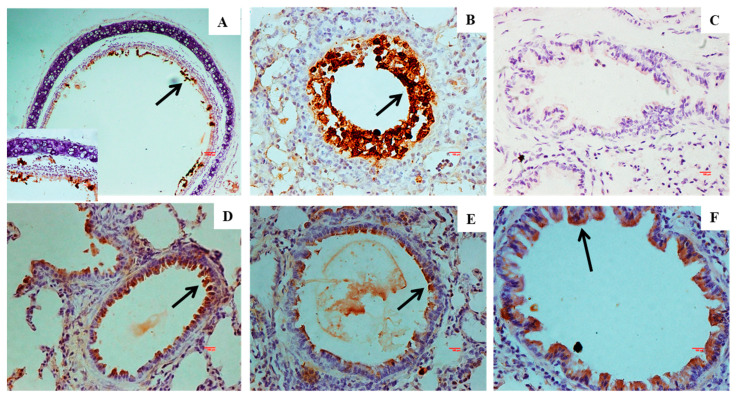
IIPT sections of trachea and lungs of mice. (**A**) PC showing strong EHV-1 immunopositivity in the tracheal epithelium with desquamated necrotic debris in the lumen at 3 dpc (IHC, 100X). Inset showing the same section (IHC, 400X); (**B**) PC showing strong EHV-1 immunopositivity in the bronchiolar epithelium at 3 dpc (IHC, 400X); (**C**) NC showing no EHV-1 immunopositivity at 9 dpc (IHC, 400X); (**D**) vRaj, (**E**) vToH-DMV, and (**F**) vToH-QMV showing EHV-1 immunopositivity in the bronchiolar epithelium (IHC, 400X).

The mice from the wt group showed similar findings, albeit with reduced intensity (Figure 7B,F,J). Lesions in the vToH-QMV and vToH-DMV groups were characterized by mild to moderate changes in terms of consolidation (Figure 7C,D), neutrophil infiltration, and necrotizing alveolitis and bronchiolitis (Figure 7G,H). Among the immunized groups, the severity of lesions was highest in the wt group, followed by the vToH-DMV and vToH-QMV groups (Figure 7K,L). Specifically, bronchiolar necrosis and cellular infiltration were less pronounced in the vToH-QMV group compared to the vToH-DMV group. Additionally, in the immunized groups, the inflammatory infiltrate mostly consisted of macrophages and fewer neutrophils. By 9 dpc, the PC group showed lesions of moderate intensity, while in the wt group, only thickening of the interlobular septa was observed in the vicinity of bronchioles and blood vessels. By 14 dpc, signs of resolution were evident in the PC group, whereas all immunized groups displayed an apparently normal interstitium. Post-challenge, the gross and histopathological scores were highest for the PC group and lowest in the vToH-QMV group (Figure 6I and Figure 7M, Table S10). In the IIPT analysis, strong EHV1 antigen expression was detected in the tracheal (Figure 8A) and bronchial epithelium (Figure 8B) of the PC group at 3 dpc. In contrast, the NC group mice showed no immunopositivity in these tissues (Figure 8C). Mild to moderate immunopositivity was also observed in the immunized groups, indicating limited viral replication in the bronchial and bronchiolar epithelium (Figure 8D–F).

### 3.5. Nucleic Acid Quantification of EHV1 Mutants Against Wt Post Challenge by qPCR

**Figure 9:**
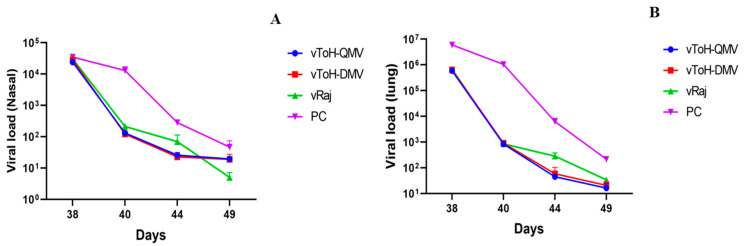
Purified viral DNA was subjected to a specific TaqMan probe-based real-time qPCR assay targeting the ORF30 gene of EHV-1, and the copy numbers were measured. (**A**) Nasal shedding of virus from different groups at different intervals; (**B**) lung viral load from different groups at different intervals.

The viral load in the nasal wash and lung tissue of mice was quantified as EHV1 genome copies using qPCR using TaqMan probe chemistry. The immunization of mice with EHV1 mutants resulted in a significant reduction in nasal virus shedding compared to the wt group, both in terms of duration and magnitude. At 7 dpv, viral copy numbers in the wt group were significantly higher than those in the mutant groups (vToH-QMV and vToH-DMV), after which the viral load progressively declined and became negligible until the challenge phase. Post-challenge, the mean EHV1 copy numbers in the PC group were 34,924.12 ± 6576.18 and 13,068.81 ± 4186.45 at 3 and 5 dpc, respectively. In the immunized groups, the wt group showed 30,608.7 ± 3017.29 and 212.41 ± 21.62 EHV1 genome copies, while the vToH-QMV and vToH-DMV groups showed 23,950.29 ± 3784.77, 131.88 ± 14.74, 28,255.4 ± 3483.34, and 121.27 ± 15.95 copies, respectively, at 3 and 5 dpc (Figure 9A, Table S11). By 5 dpc, the viral load in the immunized groups had decreased significantly compared to the mice in the PC group (*p* < 0.001). From 9 dpc onwards, virus clearance was observed in all immunized groups. Likewise, a similar pattern was observed in the lung tissues, where EHV1 nucleic acid quantification revealed the highest copy numbers in the wt group, followed by vToH-DMV and vToH-QMV at 3dpc (68, 9546.7 ± 24,602.55; 64,5470.5 ± 29,926.5, and 59,2780.6 ± 8344.26 for wt, vToH-DMV and vToH-QMV, respectively) (Figure 9B, Table S12). The viral copy numbers in the immunized groups were significantly lower compared to the PC group (59,74,948 ± 11,99,398.41) at 3 dpc (Figure 9B). No EHV1 genome was detected in the nasal wash or lung tissue of mice from the NC group.

## 4. Discussion

In the present study, we aimed to determine the safety and protective efficacy of double- and quadruple-deletion mutant EHV1 vaccine candidates against a virulent EHV1 challenge in a murine model. Our experimental design, involving an intranasal prime-boost vaccination strategy, induced optimal protective EHV1-specific antibody titers in both SN and ELISA, along with a robust rise in the IgM, IgG2b, IgG2a, and IgG1 antibody subclasses. Intranasal administration of the virus also increases the duration of protective immunity by contributing to the development of immune responses at the local site [29]. The SN results correlated better with the immune response shown by the IgG2a and IgG2b subclasses, which are known to possess neutralizing properties. The neutralizing capacity of the IgG subclass in mice infected with HSV-1 lies in IgG2a and IgG2b rather than IgG1 [30,31]. In the vToH-QMV mutant virus, deletion of the gE, IR6, pUL43, and pUL56 genes from the parent strain vToH did not affect viral replication and was highly effective in generating a humoral immune response in mice. Antibodies recognizing the exposed glycoproteins on the envelope surface of EHV1 are mostly beneficial during the early stages of infection, as they have little effect on cell-associated viremia [32]. Moreover, humoral immunity is short-lived, rendering animals more prone to reinfection after inactivated vaccine immunization [33]. An effective vaccine should induce high titers of neutralizing antibodies and elevated frequencies of cytotoxic T lymphocytes (CTL) [34]. The cytotoxic activity of CD8+ cells is believed to eliminate EHV1-infected cells from circulation, thereby restricting cell-associated viremia and reducing the likelihood of EHV1 dissemination to endothelial cells [35,36].

Mice from the wt group initially showed a significant rise in T-cell response compared to the mutant groups (vToH-QMV and vToH-DMV) at 7 dpv. However, from 14 dpv onwards, the CD8+ response significantly decreased and remained at low frequencies throughout the study. An earlier report on experimental EHV1 infection in horses revealed suppression or a strong reduction in the number of EHV1-infected lymphocytes [37]. Additionally, during a CD8+ response to infection, the majority of the activated CD8+ effector cells die by apoptosis, and the remaining antigen-specific CD8+ cells eventually form a pool of long-lived memory T-cells [38]. This could explain the drastic fall in CD8+ immunophenotype in mice infected with the wt strain. Furthermore, the pUL56 gene present in the wt virus may be responsible for MHC-1 downregulation, resulting in weak T-cell induction, causing delayed or weak T-cell responses. This downregulation might have led to the defective presentation of antigenic peptides, crippling the CTL’s ability to eliminate infected cells due to a failure to recognize antigens [39,40]. A similar decrease in the CD8+ immunophenotype was observed in the mice immunized with vToH-DMV (intact pUL56 and pUL43). This could further support the role of pUL56 in causing immunosuppression. On the other hand, the mice immunized with vToH-QMV showed a significantly less CD8+ response at 7 dpv, but no such reduction was observed thereafter. In contrast to the other immunized groups, the T-cell response of vToH-QMV increased from 14 dpv and peaked at 35 dpi. Deletion of the pUL56 gene might have improved the MHC-I expression in mice immunized with vToH-QMV, leading to enhanced antigen presentation and a stronger T-cell response. In an experimental infection of horses using virus mutants with multiple deletions, including ORF1 and ORF71, it was reported that CD8+ and INFγ-positive cells were dominant, whereas CD4+ and IFNγ-positive cells remained lower throughout the study.

Mice were challenged three weeks after the second immunization when their immune responses were at peak. The PC group was fully susceptible and severely affected upon challenge, showing an approximate 6% loss in their pre-infection body weight. Earlier studies have also reported that mice experimentally infected with EHV1 showed a reduced feed and water intake, hunched posture, dyspnoea, and ruffled fur [21,41]. In contrast, the immunized groups did not manifest clinical signs nor exhibit any weight loss post-challenge, demonstrating the lower virulence of the virus.

In this study, gross and histopathological lesions were mainly confined to the lungs. Maximum pathological changes were observed in the PC group at 3 and 5 dpc, corroborating the clinical findings and EHV1 nucleic acid quantification [10,14]. Gross lesions displayed variable degrees of congestion and red and gray hepatization. Similarly, the histopathological findings included interstitial thickening and necrosis; necrotizing bronchitis and bronchiolitis with a strong demonstration of EHV1 antigen in the IPT-stained sections; and perivascular and peribronchiolar cuffing, consistent with the findings from previous studies [17,21,42,43]. Among the immunized groups, the wt group showed more severe pathology and higher nucleic acid quantification compared to the mutant groups. Viral copy numbers were significantly lower in the mutant groups, particularly in vToH-QMV. Similar results were observed in the EHV1 homologs, where the extent of virus shedding was reduced in the animals that were intranasally inoculated with gE and gI deletion mutants [44,45,46]. Despite immunization with the wild virus, the protection it offered was not robust enough to prevent pathology, as the mice still exhibited extensive lung lesions compared to the mutant groups. This could be attributed to the inadequate CD8+ response at the time of the challenge, as well as increased neutrophil recruitment, which led to the enhanced production of proinflammatory mediators. These mediators, as a major source of reactive oxygen species, likely caused more damage to the lung tissues due to an increased inflammatory response orchestrated by the host [47,48]. The present study demonstrated similar findings, showing that gene deletion mutants were less virulent and induced immune responses comparable to or better than the parent strain [36,49,50].

Altogether, the results indicated that the challenge virus was quickly cleared from the lungs of mice immunized with EHV1 mutants (vToH-QMV and vToH-DMV). In particular, the ToH-QMV mutant effectively limited viral replication in the lungs post-challenge, correlating with the upsurge in antibody responses (primarily IgG1 and IgG2b) and enhanced CD8+ responses, leading to early restoration of clinical signs and body weight loss.

## 5. Conclusions

In mice, both EHV1 mutants (vToH-QMV and vToH-DMV) demonstrated a high level of attenuation and safety. Both vaccine viruses induced optimal levels of EHV1-specific humoral and cell-mediated immune responses, as evident by the virus neutralization assay, ELISA, and immunophenotyping. Further, after a virulent challenge with the pathogenic wild-type virus, mice immunized with vToH-QMV showed a reduced pathology in terms of their clinical signs, body weight loss, and fewer gross and histopathological lesions, along with early viral shedding from the respiratory tract. This enhanced protection can be attributed to the higher CD8+ T-cell response and elevated neutralizing antibody titers observed in these mice. Despite a high-challenge dose, the rapid recovery and protection conferred by the quadruple-deletion EHV1 mutant suggest its potential as an effective vaccine candidate for mitigating EHV1 infections in horses. Thus, vToH-QMV qualifies to be a good modified live virus (MLV) vaccine candidate for vaccinating against EHV, with the additional potential to act as a vector for developing vaccines against other equine diseases. Further testing of these vaccine candidates in the natural host, i.e., equines, is warranted, which will be carried out in our near future immunization challenge study in horses.

## Data Availability

The original contributions presented in this study are included in the article/supplementary material. Further inquiries can be directed to the corresponding author/s.

## Supplementary Materials

The following supporting information can be downloaded at: https://www.mdpi.com/article/10.3390/vaccines13010045/s1, Table S1: Experimental design for the challenge study, Table S2: Humoral immune response: Serum neutralization antibody titers of mice from different groups at different intervals, Table S3: Humoral immune response. OD values from pooled sera of mice (1:800) from different groups at different intervals, Table S4: Cellular immune response. Mean CD4% in PBMC of mice from different groups at various intervals, Table S5. Cellular immune response. Mean CD8% in PBMC of mice from different groups at various intervals, Table S6: Mean percentage body weight loss/gain in different groups of mice post first immunization, Table S7: Mean percentage body weight loss/gain in different groups of mice post second immunization, Table S8: Mean percentage body weight loss/gain in different groups of mice post-challenge, Table S9: Mean gross score for overall intensity of lung lesions at various intervals in different groups of mice, Table S10: Mean histopathological lung lesions score for overall intensity of lung lesions at various intervals in different groups of mice. Table S11: Detection of EHV1 nucleic acid in nasal wash of mice across various groups (in terms of DNA copy numbers) at different time intervals. Table S12: Detection of EHV1 nucleic acid in lungs of mice across various groups (in terms of DNA copy numbers) at different time intervals.
