# Supplementary material for: Multiple Gene Deletion Mutants of Equine Herpesvirus 1 Exhibit Strong Protective Efficacy Against Wild Virus Challenge in a Murine Model
Source: Vaccines (Basel). 2025 Jan 8;13(1):45. doi: 10.3390/vaccines13010045 (PMC11768829; doi:10.3390/vaccines13010045)
Supplement: Supplementary file 1 [file vaccines-13-00045-s001.zip › vaccines-3304174-supplementary.pdf]

Supplementary file

Table S1: Experimental design for the challenge study

**Group name**	**Grp-I (EHV1 vToH-QMV)**	**Grp-II (EHV1 vToH-DMV**	**Grp-III (EHV1-wt)**	**Control**	
	**vTOHΔIR6/UL43/gE/UL56**	**vTOHΔIR6/gE**	**vRaj**		
No of animals to be sacrificed on 0 day (pre immunization)	3				
No. of animals in each group	29	29	29	34	
Immunization	0 and 14th day			Mock immunization	
No of animals to be sacrificed/ interval post immunization	3	3	3	3	
No of intervals for sacrifices	3 (7,14,35 days post immunization)				
Challenge	35th day			Control +ve **(Grp-IV)**	Control -ve **(Grp V)**
				Challenge	Mock challenge
No of animals to be sacrificed/ interval post challenge	5	5	5	5	5 (9dpc only)
No of intervals for sacrifices	4 (3,5,9,14 days post challenge)				

Table S2: Humoral immune response: Serum neutralization antibody titres of mice across different groups and time intervals

	Days post immunization			Days post challenge			
Pre imm	-						
**Groups**	**7**	**14**	**35**	**38**	**40**	**44**	**49**
Grp IV (PC)	NA			-	-	2.7	4
Grp V (NC)	NA					-	NA
Grp I (EHV1 vToH-QMV	4	16	256	128	256	256	128
Grp II (EHV1 vToH-DMV)	8	32	128	128	256	256	256
Grp III EHV1 vRaj)	4	16	128	64	128	256	128

Table S3: Humoral immune response: OD values from pooled sera of mice (Dilution 1:800) across different groups and time intervals

**Mice on day zero - 0.06±0.00**					
**Days**	**vToH-QMV**	**vToH-DMV**	**vRaj**	**PC**	**NC**
7	0.13±0.01 $^{D\,****\,x}$	0.28±0.00 $^{a\,C\,x}$	0.02±0.01 $^{b\,x}$	0.089±0.01^{x}	-
14	0.52±0.00 $^{a\,***\,y}$	0.63±0.03 $^{a\,A\,y}$	0.50±0.00 $^{a\,y}$	0.08±0.00	-
35	1.38±0.02 $^{a\,A\,****\,y}$	1.23±0.00 $^{a\,A\,y}$	0.91±0.01 $^{a\,y}$	0.09±0.01	-
38	1.29±0.01 $^{a\,B\,y}$	1.33±0.04 $^{a\,A\,y}$	1.18±0.01 $^{a\,y}$	0.09±0.00	-
40	1.97±0.09 $^{a\,A\,****\,y}$	1.79±0.03 $^{a\,A\,y}$	1.59±0.03 $^{a\,y}$	0.16±0.00 ^{y}	-
44	2.06±0.02 $^{a\,p\,y}$	2.02±0.04 $^{a\,p\,y}$	2.03±0.01 $^{a\,p\,y}$	0.84±0.01 ^{y}	0.00±0.00 ^{a}
49	1.92±0.01 $^{a\,D\,****\,y}$	2.09±0.04 $^{a\,A\,y}$	1.84±0.03 $^{a\,y}$	1.04±0.00 ^{y}	-

$^{a\,,\,b,\,c}$ and ^{d} indicates significant difference at (p<0.0001), (p<0.001), (p<0.01) and (p<0.05), respectively, when immunized groups are compared with group IV; $^{p,\,q,\,r}$ and ^{s} indicates significant difference at (p<0.0001), (p<0.001), (p<0.01) and (p<0.05), respectively, when immunized groups are compared with negative control group; $^{A,\,B,\,C}$ and ^{D} indicates significant difference at (p<0.0001), (p<0.001), (p<0.01) and (p<0.05), respectively, when groups are compared with group III; *** indicates significant difference at (p<0.001), ** at (p<0.01) and * (p<0.05) , when groups are compared with group II; x to the y indicates significant difference across a column as determined by two way ANOVA, Dunett's test.

Table S4: Cellular immune response: Mean CD4 % in PBMC of mice acrossdifferent groups and time intervals

CD4					
**Days**	**vToH-QMV**	**vToH-DMV**	**vRaj**	**PC**	**NC**
7	58.25±7.83 $^{A\,x\,**}$	40.46±4.52 $^{a\,D\,x}$	26.47±1.19 $^{a\,x}$	65.93±6.75 ^{x}	-
14	44.14±4.08 $^{b\,B\,*}$	56.62±5.68 ^{y}	65.39±3.37 ^{y}	64.46±0.51	-
35	33.43±6.45 $^{a\,A\,y}$	44.74±6.63 $^{b\,D}$	57.08±3.44 ^{y}	64.29±3.06	-
38	47.92±3.05 ^{B}	51.73±5.12 ^{D}	62.53±0.76 $^{c\,y}$	48.90±1.85 ^{y}	-
40	46.74±4.64 $^{c\,B\,y}$	55.97±3.51 ^{y}	62.83±2.54 ^{y}	58.81±1.70	-
44	51.23±3.15 $^{D\,p}$	53.76±2.73 $^{q\,y}$	61.03±1.91 ^{y}	53.99±2.17 ^{y}	68.54±0.41^{b}
49	53.82±1.51^{d}	59.00±3.05 ^{y}	59.46±2.20^{y}	63.90±0.48	-

Each value represents mean ± SEM ^{a} , ^{b,} ^{c} and ^{d} indicates significant difference at (p<0.0001), (p<0.001), (p<0.01) and (p<0.05), respectively, when immunized groups are compared with group IV; ^{p,} ^{q,} ^{r} and ^{s} indicates significant difference at (p<0.0001), (p<0.001), (p<0.01) and (p<0.05), respectively, when immunized groups are compared with negative control group ^{A,} ^{B,} ^{C} and ^{D} indicates significant difference at (p<0.0001), (p<0.001), (p<0.01) and (p<0.05) , respectively, when groups are compared with group III; *** indicates significant difference at (p<0.001), ** at (p<0.01) and * (p<0.05), when groups are compared with group II; x to the y indicates significant difference across a column as determined by two way ANOVA, Dunett's test.

Table S5. Cellular immune response: Mean CD8 % in PBMC of mice across different groups and time intervals

CD8					
**Days**	**vToH-QMV**	**vToH-DMV**	**vRaj**	**PC**	**NC**
7	36.12±6.52 $^{A\,**\,x}$	53.41±2.98 $^{b\,D\,x}$	68.04±2.23 $^{a\,x}$	32.56±0.63 ^{x}	-
14	52.52±3.22 $^{b\,B\,y}$	40.20±5.59 ^{y}	30.83±3.16 ^{y}	32.21±0.9	-
35	63.56±6.45 $^{a\,A\,y}$	51.31±7.30 ^{b}	39.27±3.49 ^{y}	32.22±2.91	-
38	49.49±3.04 $^{B\,y}$	45.08±5.11^{D}	34.88±0.81 $^{d\,y}$	45.53±4.65 ^{y}	-
40	48.37±3.68 $^{d\,C\,y}$	41.93±3.63	34.14±2.57 ^{y}	37.79±1.43	-
44	45.89±3.13 ^{p}	43.04±3.35 ^{r}	37.21±1.87 ^{y}	44.16±2.11	29.22±0.41^{b}
49	44.34±1.49 ^{d}	39.18±2.87 ^{y}	38.44±2.12 ^{y}	33.95±0.27	-

Each value represents mean ± SEM ^{a} , ^{b,} ^{c} and ^{d} indicates significant difference at (p<0.0001), (p<0.001), (p<0.01) and (p<0.05), respectively, when immunized groups are compared with group IV; ^{p,} ^{q,} ^{r} and ^{s} indicates significant difference at (p<0.0001), (p<0.001), (p<0.01) and (p<0.05), respectively, when immunized groups are compared with negative control group ^{A,} ^{B,} ^{C} and ^{D} indicates significant difference at (p<0.0001), (p<0.001), (p<0.01) and (p<0.05), respectively, when groups are compared with group III; *** indicates significant difference at (p<0.001), ** at (p<0.01) and * (p<0.05), when groups are compared with group II; x to the y indicates significant difference across a column as determinedby two way ANOVA, Dunett's test.

Table S6: Mean percentage body weight loss/gain across different groups of mice post first immunization

Days	vToH-QMV	vToH-DMV	vRaj	PC
1	-3.59±2.63 [d]	-2.62±2.81	-3.67±3.59 [d]	2.39±4.31
2	-2.85±3.06 [b C]	-6.39±5.23 [a]	-8.74±5.86 [a]	5.68±4.16
3	0.04±3.93 [b A *]	-5.32±7.80 [a]	-9.86±7.59 [a]	8.08±5.06
4	1.25±3.29 [b A]	-3.09±9.84 [a]	-7.33±8.18 [a]	9.26±4.95
5	1.37±3.11 [a A]	-1.93±10.45 [aD]	-7.01±8.78 [a]	10.95±5.79
6	1.71±3.06 [a A]	0.26±1.012 [a B]	-7.11±8.22 [a]	10.93±5.94
7	2.09±2.79 [b A]	1.15±11.04 [a A]	-7.35±8.48 [a]	11.02±5.93
8	3.25±3.27 [b A]	1.97±10.59 [a B]	-5.92±8.45 [a]	12.16±6.13
9	3.75±3.34 [a A]	2.493±10.76 [a B]	-5.10±9.07 [a]	13.02±5.72
10	5.50±3.53 [b B]	3.41±10.28 [a D]	-2.24±9.55 [a]	14.51±6.44
11	7.45±4.46 [a B]	4.99±10.73 [a D]	-0.14±10.08 [a]	16.95±6.92
12	8.43±4.84 [b B]	6.17±10.64 [a C]	0.37±9.77 [a]	17.50±7.38
13	10.43±5.42 [b B]	7.75±10.00 [a D]	2.58±8.84 [a]	18.57±7.98
14	11.32±5.21 [c B]	9.08±10.25 [a D]	3.50±8.57 [a]	18.99±7.36

[a , b, c] and [d] indicates significant difference at ($p<0.0001$), ($p<0.001$), ($p<0.01$) and ($p<0.05$), respectively, when immunized groups are compared with control group; [A, B, C] and [D] indicates significant difference at ($p<0.0001$), ($p<0.001$), ($p<0.01$) and ($p<0.05$), respectively, when groups are compared with group III; *** indicates significant difference at ($p<0.001$), ** at ($p<0.01$) and * ($p<0.05$), when groups are compared with group II as determined by two way ANOVA, Dunett's test.

Table S7: Mean percentage body weight loss/gain across different groups of mice post second immunization

Days	vToH-QMV	vToH-DMV	vRaj	PC
1	0.22±2.67	-2.07±5.25	-2.87±4.16	2.65±1.14
2	2.42±2.64 [D]	-1.70±8.51 [d]	-3.58±6.32 [c]	4.55±1.75
3	4.30±3.39 [C *]	-0.84±9.39 [d]	-2.89±9.87 [c]	5.64±1.92
4	6.138±3.72 [C]	1.26±10.33	-0.08±6.60 [d]	6.81±2.16
5	7.572±4.33 [C]	4.84±10.20	0.85±6.48 [c]	9.15±2.43
6	8.31±4.95 [D]	4.33±9.82	3.18±6.43 [d]	9.96±2.42
7	9.08±5.35 [D]	5.81±9.98	3.80±6.58 [c]	10.96±3.21
8	10.18±6.13	5.65±10.96 [d]	5.31±6.75 [d]	11.49±3.09
9	11.86±7.45 [C]	6.86±11.39	5.22±7.93 [c]	12.53±3.83
10	14.04±7.81 [C **]	7.37±12.33 [c]	7.23±9.75 [c]	14.87±4.38
11	15.69±8.10 [B **]	8.61±11.92 [c]	7.39±10.92 [b]	16.32±4.85
12	16.91±8.10 [B **]	9.87±11.43 [c]	9.00±10.36 [b]	18.02±4.78

[a , b, c] and [d] indicates significant difference at ($p<0.0001$), ($p<0.001$), ($p<0.01$) and ($p<0.05$), respectively, when immunized groups are compared with control group; [A, B, C] and [D] indicates significant difference at ($p<0.0001$), ($p<0.001$), ($p<0.01$) and ($p<0.05$), respectively, when groups are compared with group III; *** indicates significant difference at ($p<0.001$), ** at ($p<0.01$) and * ($p<0.05$), when groups are compared with group II; x to the y indicates significant difference across a column as determined by two way ANOVA, Dunett's test.

Table S8: Mean percentage body weight loss/gain across different groups of mice post challenge

Days	vToH-QMV	vToH-DMV	vRaj	PC
1	0.64±4.18 ^{d}	1.13±2.29 ^{c}	1.44±3.25 ^{c}	-2.46±2.60
2	2.85±4.69 ^{a}	2.76±3.66 ^{a}	2.08±3.40 ^{a}	-4.38±3.51
3	4.62±3.79 ^{a}	4.11±3.54 ^{a}	3.55±4.37 ^{a}	-5.46±4.16
4	6.42±4.30 ^{a}	6.71±2.93 ^{a}	5.85±3.64 ^{a}	-4.87±4.90
5	7.80±4.52 ^{a}	7.78±3.12 ^{a}	7.60±3.68 ^{a}	-4.32±4.90
6	9.96±5.03 ^{a}	8.94±2.52 ^{a}	8.36±4.24 ^{a}	-2.04±2.94
7	12.48±4.63 ^{a}	10.08±2.24 ^{a}	10.82±6.80 ^{a}	-0.55±2.50
8	13.96±5.46 ^{a}	11.10±2.48 ^{a}	10.12±7.40 ^{a}	0.39±2.89
9	12.06±4.13 $^{a D}$	10.01±2.66 ^{a}	7.21±1.39 ^{b}	0.87±3.02
10	12.73±4.21 ^{b}	9.13±2.41 ^{d}	7.82±2.08	3.21±3.88
11	14.06±4.17 ^{b}	10.51±1.23	9.42±2.50	4.82±4.30
12	14.32±3.93 ^{c}	12.16±1.92 ^{d}	10.22±2.99	5.88±4.68
13	15.54±4.31 ^{c}	13.14±2.54 ^{d}	12.44±3.50	6.80±4.34

$^{a\ ,\ b,\ c}$ and ^{d} indicates significant difference at (p<0.0001), (p<0.001), (p<0.01) and (p<0.05), respectively, when immunized groups are compared with control group; $^{A,\ B,\ C}$ and ^{D} indicates significant difference at (P<0.0001), (p<0.001), (p<0.01) and (p<0.05), respectively, when groups are compared with group III; *** indicates significant difference at (p<0.001), ** at (p<0.01) and * (p<0.05) , when groups are compared with II; x to the y indicates significant difference across a column as determined by two way ANOVA, Dunett's test.

Table S9: Mean gross score for overall intensity of lung lesions at various time intervals across different groups of mice

Days	vToH-QMV	vToH-DMV	vRaj	PC
7	0.66±0.19 $^{d\ y}$	0.66±0.19 $^{d\ y}$	1.26±0.16 $^{a\ y}$	0±0 ^{y}
14	0±0 ^{y}	0.33±0.19 ^{y}	0.6±0.35 ^{y}	0±0 ^{y}
35	0±0 ^{y}	0±0 ^{y}	0.33±0.19 ^{y}	0±0 ^{y}
38	1.4±0.23 $^{a\ A\ x}$	1.8±0.2 $^{a\ A\ x}$	3.2±0.50 $^{a\ x}$	4.67±0.96 ^{x}
40	1.4±0.31 $^{a\ A\ ****}$	2.4±0.35 $^{a\ B\ y}$	3.2±0.12 ^{a}	4.67±0.96
44	0.4±0.12 $^{a\ B\ y}$	0.6±0.2 $^{a\ A\ y}$	1.6±0.31 $^{a\ y}$	2.67±0.19 ^{y}
49	0.2±0.12 $^{a\ y}$	0.4±0.12 $^{b\ y}$	0.6±0.2 $^{c\ y}$	1.33±0.51 ^{y}

$^{a\ ,\ b,\ c}$ and ^{d} indicates significant difference at (p<0.0001), (p<0.001), (p<0.01) and (p<0.05), respectively, when immunized groups are compared with control group; $^{A,\ B,\ C}$ and ^{D} indicates significant difference at (p<0.0001), (p<0.001), (p<0.01) and (p<0.05), respectively, when groups are compared with group III; *** indicates significant difference at (p<0.001), ** at (p<0.01) and * (p<0.05) , when groups are compared with group II; ^{x} to the ^{y} indicates significant difference across a column as determined by two way ANOVA, Dunett's test.

Table S10: Mean histopathological lung lesions score for overall intensity of lung lesions at various time intervals across different groups of mice

Days	vToH-QMV	vToH-DMV	vRaj	PC
7	1.67±0.72 $^{b D y}$	2.42±0.50 ^{a}	2.67±0.72 $^{a y}$	0±0 ^{y}
14	1.25±0.79 $^{c y}$	1.67±0.82 $^{b y}$	1.92±1.00 $^{a y}$	0±0 ^{y}
35	0.75±0.42 ^{y}	0.83±0.43 ^{y}	1.17±0.19 $^{d y}$	0±0 ^{y}
38	2.75±0.53 $^{a A x}$	3.25±0.48 $^{b Cx}$	4.2±0.43 ^{x}	4.58±0.42^{x}
40	2.1±0.96 $^{a A}$	2.75±0.34 $^{a B}$	4±0.28	4.33±0.47
44	1.25±0.62 $^{a A * y}$	2.1±0.12 $^{a C y}$	3.05±0.3 ^{y}	3.5±0.19 ^{y}
49	0.80±0.73 $^{b C y}$	1.3±0.26 ^{y}	1.8±0.28 ^{y}	2±0.47 ^{y}

$^{a\ ,\ b,\ c}$ and ^{d} indicates significant difference at ($p<0.0001$), ($p<0.001$), ($p<0.01$) and ($p<0.05$), respectively, when immunized groups are compared with control group; $^{A,\ B,\ C}$ and ^{D} indicates significant difference at ($p<0.0001$), ($p<0.001$), ($p<0.01$) and ($p<0.05$), respectively, when groups are compared with group III; *** indicates significant difference at ($p<0.001$), ** at ($p<0.01$) and * ($p<0.05$), when groups are compared with group II; ^{x} to the ^{y} indicates significant difference across a column as determined by two way ANOVA, Dunett's test.

Table S11: Detection of EHV1 nucleic acid in nasal wash of mice across various groups (in terms of DNA copy numbers) at different time intervals

Days	vToH-QMV	vToH-DMV	vRaj	PC	NC
Post immunization					
7	148.92±0 ^{D}	14.42±11.58 ^{C}	354.62±189.02 ^{x}	ND	
14	16.28±5.09	14.92±6.59	45.41±47.84 ^{y}	ND	
35	27.16±14.05	17.71±13.22	31.10±18.01 ^{y}	ND	
Post challenge					
38	23950.29±3784.34 ^{cx}	28255.4±3483.77 $^{d C}$ ^{x}	30608.7±3017.29 ^{x}	34924.12±6576.18 ^{x}	
40	131.88±14.74 $^{b y}$	121.27±15.95 $^{b y}$	212.41±21.62 $^{b y}$	13068.81±4186.45 ^{y}	
44	25.66±4.03 ^{y}	22.64±8.87 ^{y}	69.99±43.83 ^{y}	282.16±59.23 ^{y}	ND
49	19.44±0.99 ^{y}	18.99±8.49 ^{y}	5.02±2.14 $^{c y}$	46.68±27.15 ^{y}	

Each value represents mean ± SD. $^{a\ ,\ b,\ c}$ and ^{d} indicates significant difference at ($p<0.0001$), ($p<0.001$), ($p<0.01$) and ($p<0.05$), respectively, when immunized groups are compared with group IV; $^{A,\ B,\ C}$ and ^{D} indicates significant difference at ($p<0.0001$), ($p<0.001$), ($p<0.01$) and ($p<0.05$), respectively, when groups are compared with group III; ^{x} to the ^{y} indicates significant difference across a column as determined by two way ANOVA, Dunett's test

Table S12: Detection of EHV1 nucleic acid in lungs of mice across various groups (in terms of DNA copy numbers) at different time intervals

Days	vToH-QMV	vToH-DMV	vRaj	PC	NC
Post immunization					
7	45.17±7.89 ^{A}	28.53±10.78 ^{A}	1432.17±101.33 ^{x}	ND	
14	41.59±7.42	29.03±26.33	103.17±120.71 ^{y}	ND	
35	19.22±24.96	28.64±7.37	18.06±10.53 ^{y}	ND	
Post challenge					
38	592780.6±8344.26 ^{a}	645470.5±29926.5 ^{a}	689546.7±24602.55 ^{a}	5974948±1199398.41 ^{x}	
40	840.38±92.46 ^{c}	902.57±43.73 ^{c}	849.5±229.81 ^{c}	1038588±109120.76 ^{y}	
44	45.16±12.30	58.63±43.76	282.58±95.99	6240.60±2255.11 ^{y}	ND
49	16.36±8.66	20.76±8.44	34.11±6.54	215.50±13.24 ^{y}	

Each value represents mean ± SD. ^{a}, ^{b}, ^{c} and ^{d} indicates significant difference at ($p<0.0001$), ($p<0.001$), ($P<0.01$) and ($p<0.05$), respectively, when immunized groups are compared with group IV; ^{A}, ^{B}, ^{C} and ^{D} indicates significant difference at ($p<0.0001$), ($p<0.001$), ($p<0.01$) and ($p<0.05$), respectively, when groups are compared with group III; ^{x} to the ^{y} indicates significant difference (pre and post immunization) across a column as determined by two way ANOVA, Dunett's test.
